# Richness and distribution of endangered orchid species under different climate scenarios on the Qinghai-Tibetan Plateau

**DOI:** 10.3389/fpls.2022.948189

**Published:** 2022-09-07

**Authors:** Huawei Hu, Yanqiang Wei, Wenying Wang, Ji Suonan, Shixiong Wang, Zhe Chen, Jinhong Guan, Yanfang Deng

**Affiliations:** ^1^College of Geosciences, Qinghai Normal University, Xining, China; ^2^Key Laboratory of Remote Sensing of Gansu Province, Northwest Institute of Eco-Environment and Resources, Chinese Academy of Sciences, Lanzhou, China; ^3^College of Life Sciences, Qinghai Normal University, Xining, China; ^4^Key Laboratory of Biodiversity Formation Mechanism and Comprehensive Utilization in Qinghai Tibet Plateau, Xining, China; ^5^Qinghai Service and Guarantee Center of Qilian Mountains National Park, Xining, China

**Keywords:** orchid species, climate change, species richness, mean elevation, MaxEnt model, potential distribution

## Abstract

Predicting the potential influences of climate change on the richness and distribution is essential for the protection of endangered species. Most orchid species are narrowly distributed in specific habitats and are very vulnerable to habitat disturbance, especially for endangered orchid species on the Qinghai-Tibetan Plateau (QTP). In this study, we simulated the potential influences of climate change on the richness and distribution of 17 endangered orchid species on the QTP using the MaxEnt model based on the shared socioeconomic pathways scenarios (SSPs) in the 2050s and 2070s. The results showed that aspect, annual precipitation, elevation, mean temperature of driest quarter, topsoil pH (H_2_O), and topsoil sand fraction had a large influence on the potential distribution of endangered orchid species on the QTP. The area of potential distribution for orchid species richness ranging from 6 to 11 under the current climate scenario was 14,462 km^2^ (accounting for 0.56% of QTP), and it was mostly distributed in the southeastern part of QTP. The area of orchid species richness ranging from 6 to 11 under SSP370 in the 2070s was the smallest (9,370 km^2^: only accounting for 0.36% of QTP). The largest area of potential distribution for orchid species richness ranging from 6 to 11 was 45,394 km^2^ (accounting for 1.77% of QTP) under SSP585 in the 2070s. The total potential distribution area of 17 orchid species richness all increased from the 2050s to the 2070s under SSP126, SSP245, SSP370, and SSP585. The orchid species richness basically declined with the increasing elevation under current and future climate scenarios. The mean elevation of potential distribution for orchid species richness ranging from 6 to 11 under different climate scenarios was between 3,267 and 3,463 m. The mean elevation of potential distribution for orchid species richness ranging from 6 to 11 decreased from SSP126 (3,457 m) to SSP585 (3,267 m) in the 2070s. Based on these findings, future conservation plans should be concentrated on the selection of protected areas in the southeastern part of QTP to protect the endangered orchid species.

## Introduction

Orchidaceae, with more than 25,000 species and about 880 genera in the world, is one of the most diverse and largest families of flowering plants (Fay and Chase, 2009). They have the highest speciation rate, but they also have the highest extinction rate (Gravendeel et al., 2004). Most orchid species have narrow habitats and are more susceptible to habitat disturbance than other plants (Cozzolino and Widmer, 2005), which may result in large-scale extinctions with climate change (Swarts and Dixon, 2009). Orchid species are considered as the flagship group of biological protection because of their ornamental and medicinal value, endangered status, and important role in the ecosystem.

The Qinghai-Tibetan Plateau (QTP) is rich in orchid species. In addition, more than one-third of the orchid species on the QTP are endemic to China. The QTP is the highest and largest plateau in the world with an average altitude of more than 4,000 m and an area of 2.5 million km^2^ (Zhang et al., 2016). The larger latitude span makes the region have more low-latitude belts. The various topographic conditions and climate types give birth to the rich biodiversity in the low-latitude belt, which is one important hotspot of the 34 biodiversity hotspots in the world (Medail, 2001). The QTP with its diverse vegetation, complicated geographical environment, and relatively less disturbance from human activity comes into being a relatively unique natural environment and ecosystem. The two characteristic features of QTP, namely, primitiveness and fragility, make the plateau an ideal place to study the dynamic in vegetation and its reaction to climate change (Zhang et al., 2014). Compared with other plants, orchid species are extremely impressionable to climate change (Juiling et al., 2020; Liu et al., 2021). The southwestern region (Yunnan and Sichuan) is the distribution and differentiation center of orchid species compared with other areas of China (Guo and Wang, 2013).

How species richness varies along an altitude gradient has been a hot topic in biodiversity studies (Acharya et al., 2011). There are three types of relationships, namely, high richness at low altitude, hump mode with high diversity at middle altitude, and monotonically decreasing pattern with altitude (Grytnes and Beaman, 2006; Wang et al., 2007). Climatic variables are the most important factors to explain species abundance patterns with altitude, especially for large-scale research (Sanders et al., 2007). The study by Acharya et al. (2011) shows that precipitation and temperature can explain clearly the orchid species abundance along the Himalayan elevational gradient. There are several mechanisms related to the regions and environments to describe the pattern of orchid species richness along the elevational gradient in Yunnan (Zhang et al., 2015). However, there is no related study on the changes in orchid species richness along the altitude gradient on the QTP, especially predicting potential richness using the MaxEnt model under different shared socioeconomic pathways scenarios (SSPs).

The MaxEnt model, mainly used in ecology and biogeography research fields, is currently the most widely used species distribution model (Elith et al., 2006; Phillips et al., 2006; Barbosa and Schneck, 2015; Vaz et al., 2015). Research fields mainly involve potential distribution research of species under different climate change scenarios (Li et al., 2013), prediction of the area of potential distribution for invasive organisms (Callen and Miller, 2015), and suitable habitat research of endangered species and species with the economic value (Wan et al., 2014; Chemura et al., 2016). The MaxEnt model is easy to operate, can use categorical and continuous data as environmental variables to participate in modeling, and can obtain stable results even with a small amount of sample data (Phillips and Dudik, 2008). SSPs, provided by the sixth Coupled Model Intercomparison Project (CMIP6), consider the impacts of socioeconomic and land use on the development of regional climate change (Kebede et al., 2018; Zhang et al., 2019). The scenarios of SSP126, SSP245, SSP370, and SSP585 were used to predict the potential richness and distribution of orchid species. The starting point of SSPs is high, and the simulation result is close to the real value (Riahi et al., 2017).

In this study, the endangered 17 orchid species on the QTP were predicted using the MaxEnt model under different climate scenarios in the current, the 2050s, and the 2070s. Based on these databases, together with other environmental variables, we evaluated the relationship between elevation and orchid species richness under different climate scenarios on the QTP. The questions we tried to answer were as follows: (1) How orchid species richness is distributed? (2) Which environmental factors affected the distribution of orchid species richness on the QTP? and (3) How the area and mean elevation of potential distribution for orchid species richness on the QTP changed? These findings will be valuable for establishing reserve sites to protect the endangered orchid species on the QTP.

## Materials and methods

### Study area

**FIGURE 1:**
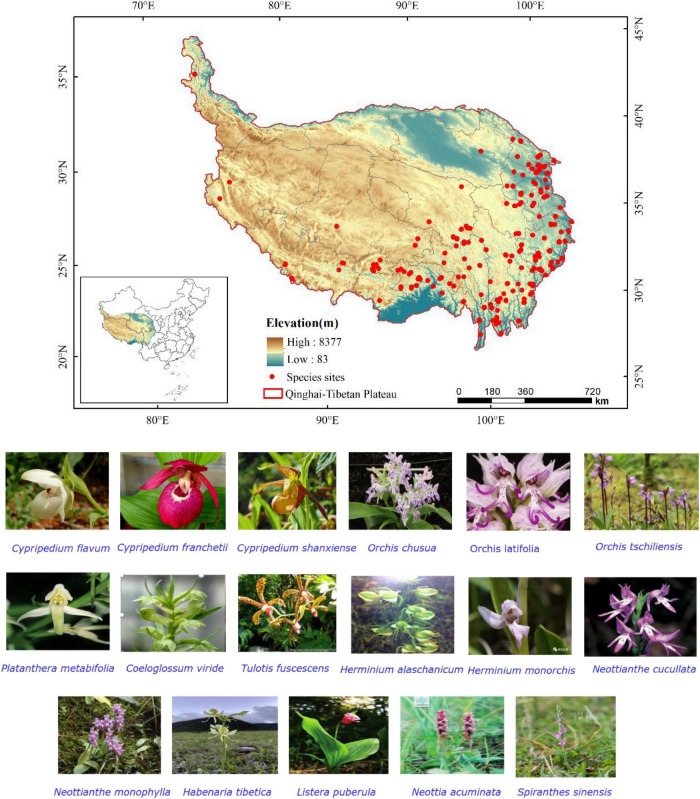
Geographical locations and photos of 17 orchid species on the Qinghai-Tibetan Plateau (QTP).

The Qinghai-Tibetan Plateau, located between 73°19′∼104°47′E and 26°00′∼39°47′N, is the highest plateau in the world with an average altitude of above 4,000 m above sea level. The total area is about 2.5 million km^2^ (Figure 1). The special geographical environment and surface features of the QTP have formed an extremely complex climatic condition (Yang et al., 2020). From southeast to northwest of the QTP presents a climate change from warm-wet to cold-dry (Feng et al., 2020). In terms of annual precipitation, there are obvious differences between seasons and regions on the QTP. The precipitation is mainly concentrated in summer, and the main distribution of precipitation in spring and autumn is in the southern QTP. The QTP become the region with the strongest climate change in the context of global warming. Due to the fragile and vulnerable climate change, the QTP is an ideal region to study the impacts of global warming on the alpine vegetation system (Xu et al., 2016).

### Study species

The orchid species were selected from the list of national key protected and endangered species. They have great medical or ornamental value and have more distribution sites. Finally, the 17 orchid species on the QTP selected in this study are as follows: *Cypripedium flavum*, *Cypripedium franchetii*, *Cypripedium shanxiense*, *Orchis chusua*, *Orchis latifolia*, *Orchis tschiliensis*, *Platanthera metabifolia*, *Coeloglossum viride*, *Tulotis fuscescens*, *Herminium alaschanicum*, *Herminium monorchis*, *Neottianthe cucullate*, *Neottianthe monophyla*, *Habenaria tibetica*, *Listera puberula*, *Neottia acuminata*, and *Spiranthes sinensis*. Among them, *C. flavum*, *C. franchetii*, *O. tschiliensis*, *N. monophyla*, and *H. tibetica* are endemic to China. The 17 orchid species included vulnerable (VU), near threatened (NT), and endangered (EN) species (Table 1; IUCN, 2019). The main value, the red list level, and the protected level of 17 orchid species were obtained from China Rare and Endangered Plant Information System.^1^ The geographical location information for 17 endangered orchid species from nine genera was obtained from the following: (1) Global Biodiversity Information Facility (GBIF^2^) and (2) Chinese Virtual Herbarium (CVH^3^). Species distribution records from 1864 to 2019 were selected and proofread by Google Earth,^4^ and the duplicate records were removed. The records of the occurrence data in Excel were converted to csv format for predicting.

**TABLE 1:** The 17 orchid species selected in this study.

Species name	Main values	Red list level	Protected level	Number of records
*Cypripedium flavum*	Esthetic	VU	**|**	52
*Cypripedium franchetii*	Esthetic	VU	**|**	31
*Cypripedium shanxiense*	Esthetic	VU	**|**	25
*Orchis chusua*	Esthetic	NT	**‖**	42
*Orchis latifolia*	Esthetic	NT	**‖**	60
*Orchis tschiliensis*	Esthetic	NT	**‖**	26
*Platanthera metabifolia*	Esthetic	NT	**‖**	35
*Coeloglossum viride*	Esthetic	NT	**‖**	152
*Tulotis fuscescens*	Medicinal	EN	**‖**	22
*Herminium alaschanicum*	Medicinal	NT	**‖**	75
*Herminium monorchis*	Medicinal	NT	**‖**	36
*Neottianthe cucullata*	Esthetic and medicinal	VU	**‖**	51
*Neottianthe monophylla*	Esthetic	NT	**‖**	22
*Habenaria tibetica*	Medicinal	NT	**‖**	28
*Listera puberula*	Esthetic	NT	**‖**	51
*Neottia acuminata*	Esthetic	NT	**‖**	47
*Spiranthes sinensis*	Medicinal	NT	**‖**	41

VU, vulnerable; NT, near threatened.

### Environmental variables

Initial environmental variables with 19 bioclimatic variables, three topographic variables, and 17 topsoil factors were used in this study. Bioclimatic variables were downloaded from the WorldClim dataset (Fick and Hijmans, 2017).^5^ The website provides 19 climate variables with 1 km spatial resolution related to precipitation and temperature from 1970 to 2000, which were used as baseline Climate Scenario Data. Future bioclimatic data including four scenarios of Shared Socioeconomic Pathways (SSPs) with 1 km spatial resolution were obtained from BCC_CSM2_MR provided by the sixth Coupled Model Intercomparison Project (CMIP6) (Kebede et al., 2018). SSP126, SSP245, SSP370, and SSP585 were used to analyze the spatial and temporal changes in the annual temperature and precipitation during 2041–2060 (2050s) and 2061–2080 (2070s) (Wu et al., 2019). SSP126 with (SSP1)-RCP2.6 forcing at a low level of greenhouse gas emissions is a sustainable development path; SSP245 with (SSP2)-RCP4.5 forcing at a mediate level of greenhouse gas emissions is a medium development path; SSP370 with (SSP3)-RCP7.0 forcing at a mediate-high level of greenhouse gas emissions is a medium-high development path; and SSP585 with (SSP5)-RCP8.5 forcing at a high level of greenhouse gas emissions is a development path ruled by fossil fuels (Riahi et al., 2017; Li et al., 2020).

Terrain variables including elevation, slope, and aspect were derived from the digital elevation model (DEM) by spatial analyst tools using ArcGIS10.7. The DEM data were obtained from the WIST Geodatabase of NASA.^6^ Soil factors were extracted from Harmonized World Soil Database (HWSD^7^), which provides detailed spatial information about basic soil attributes.

All environment variables (bioclimatic, topographic, and soil) were resampled to the spatial resolution of 1 km and were processed to the same geographic range. In addition, the correlation coefficient between variables was calculated to consider the effects of collinearity on the model accuracy. The variables with *r* below 0.8 and contributing larger to the model were used in the modeling (Zeng et al., 2016; Yan et al., 2020). Finally, 14 environmental variables were used in the modeling for further analysis (Table 2).

**TABLE 2:** Environmental variables selected in the model.

Data source	Variable category	Variable name	Abbreviation	Unit
WorldClim	Bioclimatic	Temperature seasonality (standard deviation * 100)	Bio4	°C
		Max temperature of warmest month	Bio5	°C
		Mean temperature of wettest quarter	Bio8	°C
		Mean temperature of driest quarter	Bio9	°C
		Mean temperature of warmest quarter	Bio10	°C
		Annual precipitation	Bio12	mm
		Precipitation of wettest month	Bio13	mm
DEM	Topographic	Elevation	Ele	m
		Slope	Slo	°
		Aspect	Asp	°
HWSD	Soil	Topsoil sodicity (ESP)	T_ESP	%
		Topsoil organic carbon	T_OC	% weight
		Topsoil pH (H2O)	T_PH_H_2_O	-log(H+)
		Topsoil sand fraction	T_SAND	%wt

### MaxEnt model processing

The MaxEnt model was used to predict the species potential distribution based on current geographic locations and associated environmental variables and provide a spatial representation of habitat suitability on a scale from 0 (lowest suitability) to 1 (highest suitability) (Shi et al., 2021). The distribution data of orchid species and 14 environmental data were imported into MaxEnt3.4.4, which was kept with other settings as default (500 iterations, 0.00001 convergence threshold, 10,000 max background point) (Elith et al., 2011). The jackknife was turned on to evaluate the importance of environmental variables (Pearson et al., 2007); 70% of the known distribution points were randomly used for training, and 30% of the distribution points were selected for testing (Jose and Nameer, 2020). The model trained with current climate data was projected on future climate data for four SSP scenarios to determine potential distributions in the 2050s and 2070s. Average output (based on ten replicates for each species) was used for further analyses. The area under the receiver operating characteristics curve (AUC) with values from 0 to 1 was used to assess the accuracy of the model. The average AUC scores from 0.7 to 0.8 are “fair,” from 0.8 to 0.9 are “good,” and more than 0.9 are “excellent.” In a word, the average AUC values of more than 0.75 are acceptable (Elith et al., 2006; Fourcade et al., 2014).

### Analysis of model predictions

**FIGURE 2:**
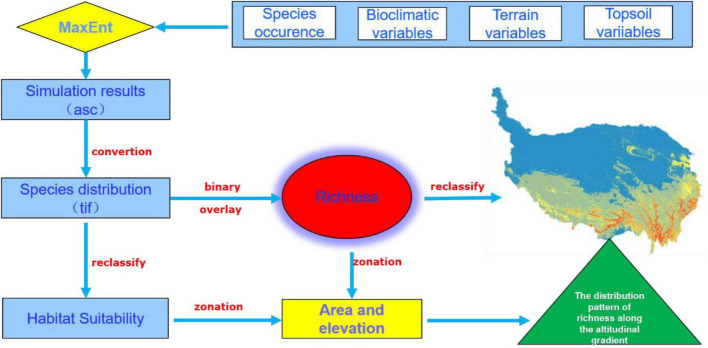
Flow diagram of the methodology adopted.

The model outputs were converted into raster format and were reclassified into four arbitrary habitat suitability categories based on nature breaks through ArcGIS (Peterson and Cohoon, 1999). The area and mean elevation of the unsuitable habitats, low suitable habitats, medium suitable habitats, and high suitable habitats were calculated by the zonal statistic tool (Brown, 2014). The richness of orchid species was obtained through binarization and superposition processing by a raster calculator in the ArcGIS platform (Figure 2). The threshold of 0.05 was selected to convert the continuous habitat suitability for each species to the binarized habitat. According to the value of richness, we divided a four-level grade scale (1, 2–3, 4–5, 6–11) to reflect the trend of orchid distribution.

## Results

### Model accuracy and contribution of environmental factors

The average AUC values of training in model with current and future climate scenarios were more than 0.9, and the mean AUC values of testing in model with current and future climate scenarios were more than 0.85. The model performance was good–excellent.

The internal jackknife test was used to evaluate the importance of environmental variables. The results showed that the distribution of 17 orchid species on the QTP was related to topography (aspect and elevation), climate (annual precipitation and mean temperature of the driest quarter), and soil [Topsoil pH (H_2_O) and Topsoil sand] (Table 3). Aspect was the most important variable influencing the distribution of the orchid species. Aspect contributed more than 50% to model output for 17 species. The following variables determining the distribution of 17 orchid species were annual precipitation (Bio12: average for 8.98%), elevation (Ele: average for 8.96%), topsoil pH (H_2_O) (T_PH_H_2_O: average for 6.49%), mean temperature of the driest quarter (Bio9: average for 4.47%), and topsoil sand fraction (T_SAND: average for 1.45%).

**TABLE 3:** The contribution (%) of environmental variables to the MaxEnt model output of 17 orchid species.

Species name	Aspect	Bio12	Elevation	Bio9	T_PH_H_2_O	T_SAND
*Cypripedium flavum*	54.5	14.9	11.3	8.8	1.2	1.1
*Cypripedium franchetii*	66.4	9.5	2.6	8.4	4.3	0
*Cypripedium shanxiense*	63.6	9.4	1.3	2	3.9	3.8
*Orchis chusua*	50.4	11.4	16.8	9.1	5.4	1.7
*Orchis tschiliensis*	63	1.7	9.4	1.4	13.5	3.6
*Neottianthe cucullata*	61.7	7.3	2.9	5.8	3.5	0.1
*Coeloglossum viride*	63.7	10.1	4.3	8.4	3.3	0.5
*Tulotis fuscescens*	51.1	2.9	20.2	1.7	17.8	1.2
*Herminium alaschanicum*	53.8	12.8	19.1	2.5	5.1	0.2
*Herminium monorchis*	64.5	8	5	3.7	8.3	1.9
*Neottianthe cucullata*	70	7.3	2.9	5.8	3.5	0
*Neottianthe monophylla*	60.2	12.5	16.9	2.1	0.1	0.1
*Habenaria tibetica*	56.2	3.7	0.6	1.9	16.8	7.4
*Listera puberula*	68.4	7.4	1.7	2.1	11.6	0.6
*Neottia acuminata*	54.2	14.3	15.4	2.5	2.9	0.7
*Spiranthes sinensis*	57.8	10.4	13.1	5.3	2.7	0.3

### Potential distribution of 17 orchid species in the current climate

The smallest area in high suitable habitat for *S. sinensis* was 18 km^2^ and concentrated in the southeastern part of Tibet. The largest area in high suitable habitat for *Neottianthe monophylla* was 174,178 km^2^ and concentrated in Tibet, Yunnan, and Sichuan. The smallest area of total suitable habitat for *T. fuscescens* was 5,291 km^2^ (only accounting for 0.21% of QTP). The largest area of the total suitable habitat for *T. fuscescens* was 1,230,403 km^2^ (accounting for 47.88% of QTP). The mean elevation in high suitable habitat for 17 orchid species was basically above 3,000 m except *T. fuscescens* and *C. flavum*. The lowest mean elevation in high suitable habitat was 678 m for *T. fuscescens.* The highest mean elevation in high suitable habitat was 4,466 m for *O. tschiliensis* (Table 4).

**TABLE 4:** The area and elevation in potentially suitable habitat for 17 orchid species under current climate conditions.

Species name	Area (km^2^) of the different habitat suitability				Mean elevation (m) of the different habitat suitability		
	High	Medium	Low	Total	High	Medium	Low
*Cypripedium flavum*	1,158	97,164	250,172	348,494	2,993	3,313	4,189
*Cypripedium franchetii*	1,233	1,545	202,241	205,019	3,357	3,508	3,414
*Cypripedium shanxiense*	1,038	2,287	618,211	621,536	3,428	3,725	4,192
*Orchis chusua*	5,275	4,118	330,769	340,162	3,269	3,192	3,750
*Orchis latifolia*	3,849	27,370	61,939	93,158	3,484	3,613	3,863
*Orchis tschiliensis*	25	1,223	14,416	15,664	4,466	3,089	4,205
*Platanthera metabifolia*	871	9,894	7,012	17,777	3,088	4,055	3,272
*Coeloglossum viride*	17,476	169,257	440,785	627,518	3,296	3,356	3,945
*Tulotis fuscescens*	30	761	4,500	5,291	678	534	608
*Herminium alaschanicum*	8,962	242,204	509,535	760,701	3,576	3,694	4,168
*Herminium monorchis*	2,339	6,050	1,016,801	1,025,190	3,420	3,446	4,216
*Neottianthe cucullata*	3,968	78,085	394,444	476,497	3,734	3,686	3,991
*Neottianthe monophylla*	174,178	415,797	601,337	1,191,312	3,348	4,155	4,493
*Habenaria tibetica*	7,373	9,788	4,688	21,849	3,725	3,486	2,828
*Listera puberula*	6,339	28,883	1,195,181	1,230,403	3,118	4,109	4,231
*Neottia acuminata*	2,111	34,030	475,179	511,320	4,090	4,141	3,836
*Spiranthes sinensis*	18	1,296	7,452	8,766	4,274	1,685	3,067

### Area changes of potential distribution for orchid species with different richness

**FIGURE 3:**
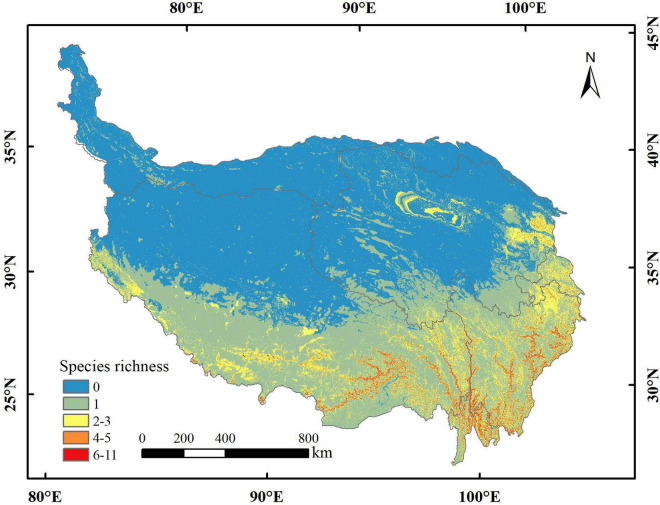
The current richness distribution of 17 orchid species on the Qinghai-Tibetan Plateau (QTP).

**FIGURE 4:**
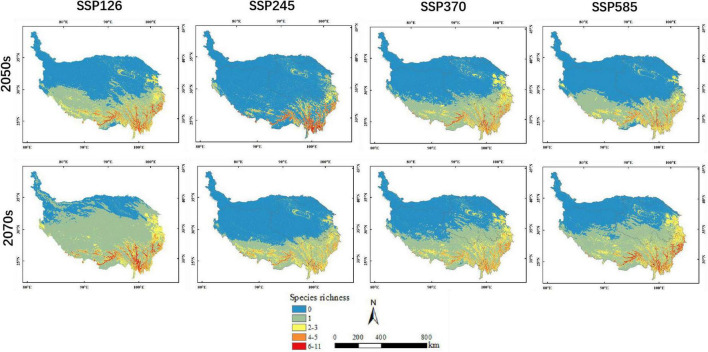
Prediction results for potential orchid species richness under different climate scenarios in the 2050s and 2070s.

The area of potential distribution for orchid species richness ranging from 6 to 11 under the current climate scenario was 14,462 km^2^ (accounting for 0.56% of QTP) and mostly distributed in the southeastern part of QTP (Figure 3). The total potential distribution area of orchid species under the current climate scenario was 1,223,314 km^2^ (accounting for 47.60% of QTP). The potential distribution area of orchid species decreased with increasing abundance under different climate scenarios (Table 5). The area of orchid species richness ranging from 6 to 11 under SSP370 in the 2070s was the smallest (9,370 km^2^: only accounting for 0.36% of QTP). The largest area of potential distribution for orchid species richness ranging from 6 to 11 was 45,394 km^2^ (accounting for 1.77% of QTP) under SSP585 in the 2070s. The largest total potential distribution area of 17 orchid species richness was 2,045,072 km^2^ (accounting for 79.57% of QTP) under SSP126 in the 2070s. The area of potential distribution for orchid species richness ranging from two to three increased from SSP126 to SSP585 in the 2070s. However, the area of potential distribution for orchid species richness ranging from four to five decreased from SSP126 to SSP585 in the 2050s. The total potential distribution area of 17 orchid species richness all increased from the 2050s to 2070s under SSP126, SSP245, SSP370, and SSP585, respectively (Figure 4).

**TABLE 5:** The area (km^2^) of orchid species richness under different climate scenarios.

Richness	2050s				2070s				Current
	SSP126	SSP245	SSP370	SSP585	SSP126	SSP245	SSP370	SSP585	
1	830,193	248,201	759,387	706,380	1,667,187	620,161	931,855	1,055,822	869,490
2–3	257,256	127,826	232,842	190,075	232,748	260,256	274,767	304,024	263,234
4–5	85,416	77,128	76,556	69,528	107,505	71,573	90,809	77,923	76,128
6–11	37,062	40,562	20,849	13,493	37,632	11,862	9,370	45,393	14,462
Total	1,209,927	493,717	1,089,634	979,476	2,045,072	963,852	1,306,801	1,483,162	1,223,314

### Mean elevation changes of potential distribution for orchid species with different richness

The mean elevation of potential distribution for orchid species richness decreased with increasing abundance under different climate scenarios except for SSP245 in the 2050s (Table 6). In a word, the orchid species richness basically decreased with ascending elevation. The mean elevation of potential distribution for orchid species richness ranging from 6 to 11 under different climate scenarios was between 3,267 and 3,463 m. The mean elevation of potential distribution for orchid species richness ranging from 6 to 11 decreased from SSP126 (3,457 m) to SSP585 (3,267 m) in the 2070s. However, the mean elevation of potential distribution for orchid species richness ranging from 2 to 3 increased from SSP126 (3,769 m) to SSP585 (3,850 m) in the 2070s. The mean elevation of potential distribution for orchid species richness ranging from 2 to 3 gradually decreased from SSP126 (3,899 m) to SSP370 (3,683 m) in the 2050s. However, the mean elevation of potential distribution for orchid species richness ranging from 2 to 3 gradually increased from SSP126 (3,769 m) to SSP370 (3,814 m) in the 2070s. The mean elevation of potential distribution for orchid species richness ranging from 4 to 5 gradually decreased from SSP126 to SSP370 both in the 2050s and 2070s.

**TABLE 6:** The mean elevation (m) of orchid species richness under different climate scenarios.

Richness	2050s				2070s				Current
	SSP126	SSP245	SSP370	SSP585	SSP126	SSP245	SSP370	SSP585	
1	4,557	3,948	4,422	4,507	4,666	4,333	4,522	4,585	4,498
2–3	3,899	3,687	3,683	3,722	3,769	3,813	3,814	3,850	3,812
4–5	3,469	3,424	3,381	3,498	3,579	3,511	3,395	3,417	3,452
6–11	3,333	3,463	3,363	3,359	3,457	3,352	3,275	3,267	3,353

## Discussion

### Potential distribution of orchid species richness

The orchid family is one of the richest in the flowering plants realm and includes many rare, threatened, and endangered species (Tsiftsis et al., 2008). In this study, we explored the richness of 17 endangered orchid species on the QTP under different climate scenarios. The results indicated that the orchid species richness is highest in the southeastern part of QTP. The richness of orchid species basically declined with the increasing elevation under the current and future climate scenarios. The relationship between species abundance and elevation has been a controversial topic in biogeography research. The previous study indicated that the highest species richness locates at the mid-elevational belts with richness declining both at higher and lower elevations (McCain, 2004). In this study, the results showed that the mean elevation of potential distribution for orchid species richness ranging from 6 to 11 under different climate scenarios was between 3,267 and 3,463 m. The potentially suitable habitats of orchid species declined above 3,500 m may be due to a change from broadleaf forest to a dominance of coniferous forest above 3,000 m (Acharya et al., 2011). The previous study found that the distribution of the orchid species was in fact a clear linear decreasing trend with elevation (Roberts and Brummitt, 2006; Timsina et al., 2021). Their results demonstrated that all species may distribute at a given elevation or an elevational range, but species richness peaked at the mid-elevation zone. In addition, the relatively low orchid species richness at low elevation may be as a result of the few intact suitable habitats that remain and extinctions due to human disturbances (Strasberg et al., 2005). Especially, 17 endangered orchid species selected in this study are more vulnerable to human effects at low altitude.

### Influences of environment variables on the potential distribution of orchid species richness

The orchid species are easily susceptible to the environment, and their potential distributions are highly related to environmental variables (Ye et al., 2020). Previous studies demonstrated that climate and soil factors could have influences on the orchid species distribution (Tsiftsis et al., 2008; Gaskett and Gallagher, 2018; Bell et al., 2021). In this study, we explored the influences of climate change, topographical factors, and soil variables on the endangered orchid species on the QTP. We found that aspect, annual precipitation, elevation, topsoil pH (H_2_O), mean temperature of the driest quarter, and topsoil sand fraction had a large influence on the distribution of endangered orchid species on the QTP, which was in accordance with the findings of previous studies (Chapagain et al., 2021).

Climate change was an important factor affecting the potential distribution of orchid species on the QTP. There were significant influences of the annual precipitation (Bio12) and mean temperature of the driest quarter (Bio9) on the potential distribution of orchid species, which may be associated with the growing elevation of different orchid species because elevation shows complicated climate factors, such as humidity and temperature (Tsiftsis et al., 2008). The orchid species distribution was found to be mainly climate driven because the total gain of the MaxEnt model was largely influenced by temperature and precipitation (Shrestha et al., 2021). Most orchid species survive in a specific temperature range, and some can tolerate temperature as low as 4°C (Acharya et al., 2011). The thermoeconomic indicator was introduced to be a link between climate change and the earth’s global temperature variation, which clarified the effect of the increasing water vapor in the atmosphere because of the rising mean earth temperature and the saturation pressure of water vapor itself (Lucia et al., 2021).

Aspect was an important factor affecting the potential distribution and richness of endangered orchid species on the QTP. The importance of aspect in the orchid distribution may be due to the more climate types. Many orchid species do not face the south slope (Shaw, 1984).

Some previous studies demonstrated that many orchid species have a symbiotic relationship with mycorrhizal fungi which depends on some soil conditions, such as nutrients, organic content, and pH (McCormick and Jacquemyn, 2014). In this study, we found that topsoil pH (H_2_O) was also one of the environment variables influencing the distribution of orchid species, which is in line with the findings of Liu et al. (2021). This may be partly because soil variables may affect the distribution of mycorrhizal fungi and have an effect on the potential distribution of orchid species.

### Influences of other factors on the potential distribution of orchid species richness

Apart from climate conditions, topographical factors and soil variables, there may be other factors affecting the distribution of orchid species, such as vegetation (Opedal et al., 2015), human disturbance (Liu et al., 2021), presence of pollinators (Duffy and Johnson, 2017), and mycorrhizal fungi (McCormick et al., 2018), which were beyond our analysis. Biotic interactions play an important role in species distribution models. If biotic interactions are not considered, it may overestimate the potential distributions of species. Almost totally orchid species depend on mycorrhizal symbionts and pollinators. The sexually deceptive orchid species are often highly specialized, so the interactions with their pollinators are expected to strongly affect distribution predictions (Tsiftsis and Djordjević, 2020). Global warming will not only cause changes in the potential distribution of orchid species but also lead to the decrease in areas suitable for pollination of orchid species. The lack of pollen vector may be devastating for fringed hare orchid which is not autogamous and must rely on a single pollinator species (Kolanowska et al., 2021). Therefore, future studies are needed to combine these factors to explore the influences on the endangered orchid richness on the QTP.

### Conservation of orchid species richness on the Qinghai-Tibetan Plateau

Orchidaceae is an important flagship group in biological protection (Pillon and Chase, 2007; Tsiftsis et al., 2019). Consistent with the condition in other areas all over the world, a large number of orchid species, especially endangered species, were driven to extinction because of overcollection, habitat fragmentation, and destruction (Swarts and Dixon, 2009). Many orchid species have high ornamental, medicinal, and other economic value, so the trade of orchid species exists in different countries and regions around the world, especially in some orchid distribution hotspots, which has been the important reason why many orchid species are endangered (Phelps and Webb, 2015). All the wild orchid species were listed in the Convention on International Trade in Endangered Species of Wild Fauna and Flora (CITES). In the past years, 17 orchid species have been evaluated according to the regional conservation status based on IUCN rank. However, some species with distribution area sizes, such as *T. fuscescens* and *S. sinensis*, were not further assessed due to their limited distributional information. Under this circumstance, *in situ* conservation is very significant to conserve threatened orchid species (Juiling et al., 2020). According to our study, the highest richness of endangered orchid species is located in the southeastern part of QTP. We need further research to identify conservation gaps and design nature reserves for protecting the endangered orchid species from extinction in these regions.

## Conclusion

In this study, we explored the potential effects of climate change on the richness and distribution of 17 endangered orchid species on the Qinghai-Tibetan Plateau based on the SSP scenarios in the 2050s and 2070s using the MaxEnt model. First, the results showed that aspect, annual precipitation, elevation, topsoil pH (H_2_O), mean temperature of the driest quarter, and topsoil sand fraction had a large influence on the potential distribution of endangered orchid species on the QTP. Second, the mean elevation of potential distribution for orchid species richness ranging from 6 to 11 under different climate scenarios was between 3,267 and 3,463 m. Third, the orchid species richness is largest in the southeastern part of QTP and basically declined with the increasing elevation under current and future climate scenarios. Based on these findings, future conservation plans should be concentrated on the selection of protected areas in the southeastern part of QTP to protect the endangered orchid species.

## Data availability statement

The original contributions presented in this study are included in the article/supplementary material, further inquiries can be directed to the corresponding authors.

## Author contributions

HH contributed to the main data search, processing, and manuscript writing work. YW and WW proposed the manuscript ideas and carried out the manuscript revision work. JS and ZC gave valuable comments in writing the manuscript and helped to collect and check the data. SW, YD, and JG carried out the manuscript revision work. All authors have read and agreed to the published version of the manuscript.

## Footnotes

1
http://www.iplant.cn/rep/protlist

2
http://www.gbif.org/

3
https://www.cvh.ac.cn/

4
http://ditu.google.cn/

5
http://www.worldclim.org

6
http://srtm.csi.cgiar.org/

7
http://www.fao.org/soils-portal
